# Evaluation of image filters for their integration with LSQR computerized tomography reconstruction method

**DOI:** 10.1371/journal.pone.0229113

**Published:** 2020-03-03

**Authors:** Mónica Chillarón, Vicente Vidal, Gumersindo Verdú

**Affiliations:** 1 Department of Computer Systems and Computation, Universitat Politècnica de València, Valencia, Spain; 2 Department of Chemical and Nuclear Engineering, Universitat Politècnica de València, Valencia, Spain; St. Vincent Medical Center, UNITED STATES

## Abstract

In CT (computerized tomography) imaging reconstruction, the acquired sinograms are usually noisy, so artifacts will appear on the resulting images. Thus, it is necessary to find the adequate filters to combine with reconstruction methods that eliminate the greater amount of noise possible without altering in excess the information that the image contains. The present work is focused on the evaluation of several filtering techniques applied in the elimination of artifacts present in CT sinograms. In particular, we analyze the elimination of Gaussian and Speckle noise. The chosen filtering techniques have been studied using four functions designed to measure the quality of the filtered image and compare it with a reference image. In this way, we determine the ideal parameters to carry out the filtering process on the sinograms, prior to the process of reconstruction of the images. Moreover, we study their application on reconstructed noisy images when using noisy sinograms and finally we select the best filter to combine with an iterative reconstruction method in order to test if it improves the quality of the images. With this, we can determine the feasibility of using the selected filtering method for our CT reconstructions with projections reduction, concluding that the bilateral filter is the filter that behaves best with our images. We will test it when combined with our iterative reconstruction method, which consists on the Least Squares QR method in combination with a regularization technique and an acceleration step, showing how integrating this filter with our reconstruction method improves the quality of the CT images.

## Introduction

In the field of health sciences, the advances in computer science and technology have allowed medicine to improve in aspects that before would have been unthinkable. For instance, the invention of magnetic resonances (MRI) or computerized tomographies (CT). Therefore, it can be said that the quality of life has been improved thanks to technology and the use of information technology as an advanced scientific instrumentation for medical applications. One of the breakthrough that we can highlight in this regard is the improvement and optimization of images for medical diagnostic purposes.

The disadvantage of the CT scanning process is the ionizing radiation used to project a body part. Each type of tissue absorbs a greater or lesser amount of radiation depending on its density. Thus, they appear in different tonalities and we can differentiate muscle tissue, bone, elements of the cardiovascular system, etc., in the images. The problem lies in the amount of radiation necessary to obtain images of acceptable resolution and sharpness. Taking a single X-ray test as a reference, a CT scanner can entail a radiation dose hundreds of times higher. In the particular case of a thorax image, the effective radiation for obtaining it by a CT scan is approximately 400 times greater than the dose necessary for the same result with a single X-ray [1].

From all of the above arises the need to reduce the effective radiation dose. This means reducing the dose for the generation of the images, which results in less exposure of the patient, but also a lower definition of the image.

Until a few years ago, image reconstruction methods based on filtered back-projection were the more commonly used mainly because they can be executed on older computers, since they have a low computational cost and the reconstruction can be done in a relatively low time. In turn, the quality of the resulting images is more than correct when a high number of projections is used, a situation in which the patient absorbs a high dose of radiation. However, the quality of the images gets worse when the number of projections is reduced.

That is why the algebraic methods of approximation started to be applied to reconstruct CT images, such as the LSQR (Least Squares QR) algorithm. They are capable of working with fewer views, as we have shown in our previous works [2–5]. But working with less projections also means more noise in the images.

In studies about Gaussian and Speckle in CT images, MRI or Ultrasounds [6, 7], the filtering techniques and algorithms proposed are applied to an already reconstructed image. Nevertheless, they could also be applied to the projection data. Therefore, the main goal of this paper is to find the most appropriate filter that can be applied either to sinograms or on CT images, so we can improve the quality of our CT reconstructions. In particular, we aim to select a filter that can be combined with an iterative reconstruction method (LSQR) as an additional step on the reconstruction algorithm when we use few projections, reducing the image noise at the same time we reduce the radiation dose.

We will explain the phases of our CT reconstruction process in Section, and also describe the chosen image filters. In Section we analyze the behavior of the filters when Gaussian and Speckle noise is added on a sinogram with few projections, as well as the subsequent evaluation of the reconstructions obtained when using the filtered sinograms. In addition, In Section we will test the filters on phantom images with added noise to see if we can improve the final result. In Section we show the results of combining the best filter with our few-view reconstruction method. We conclude with Section where we sum up the obtained results.

## Materials and methods

### CT image reconstruction process

**Fig 1:**
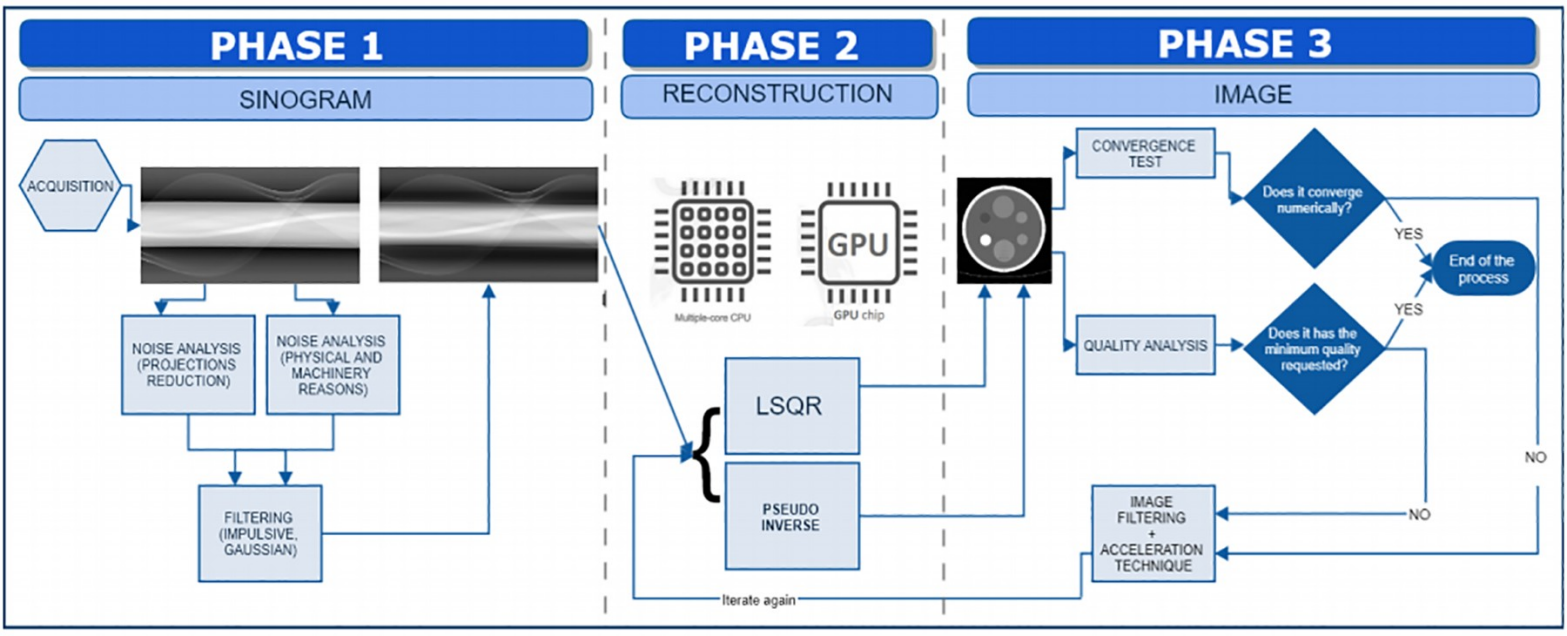
Complete CT image reconstruction process. Process of reconstructing a CT image, showing the steps from the acquisition to the final image.

When the sinograms are acquired and few views (projections) are used in the reconstruction phase of the image, artifacts of the stair-step type [8] and Gaussian and/or impulsive noise might appear. Fig 1 shows the complete process of reconstructing a CT image in two dimensions.

Phase 1 consists of the acquisition (or generation if we simulate it) of the sinogram. In this step we also study and try to eliminate the noise that may be present in the data.

In phase 2, called the algebraic reconstruction phase of the CT image, we perform the resolution of the equations system that models the CT, *Af* = *g* + *w*, where $A=(a_{i,j})\in R^{MxN}$ is the system matrix, $g\in R^{M}$ the projections vector, $w\in R^{M}$ the noise vector and $f\in R^{N}$ the desired image. The dimension *M* is the product of the number of detectors that the CT scanner has (in our case 1025) multiplied by the number of projections or views taken. *N* denotes the resolution of the image (128×128 pixels, 256×256 pixels, etc). This phase has been analyzed in previous works [2–5, 9], using several different methods to solve the equations system. Nevertheless, in those works, the approach did not include an image filter combined with the iterative process of resolution, which we will study in this paper.

Last, in phase 3, the quality of the image obtained in phase 2 is analyzed and it is decided how to proceed. We can measure the quality by image quality metrics (such as Peak Signal-to-Noise ratio) or iterate until we reach a desired relative residual *norm*(*g* − *Af*)/*norm*(*g*) (usually 10^−6^). Both in phase 1 (the generation of the sinogram) and in phase 3 (improvement of the image), image filters must be used to reduce noise, which is the main task on which the present work focuses.

To carry out the proposed studies, it is necessary to generate the system matrix *A*, and the projection vector *g*. Both data sets will have to be generated once for each desired image resolution, which has been done by the Joseph Forward Projection method [10]. This method calculates the weight of each ray on each pixel (forming the matrix *A*) weighted by the corresponding coefficients of linear attenuation of the rays. For this simulation, the Forbild Head Phantom [11] phantom was used, which mathematically represents an approximation of the existing structures in a human head. We also use an abdomen CT image selected from the dataset DeepLession [12] to test the quality of the reconstructions.

The sinogram vector *g* is formed selecting the desired projections angles from the complete generated projections. Depending on the number of views used, the amount of noise induced will vary. For these simulations, we modeled a fan-beam axial scanner.

### Iterative reconstruction method

The reconstruction method (phase 2 and 3) that is used consists of three processes that are repeated until convergence is achieved: a first process that solves the equations system. After this, an approximate solution image is obtained. Then a filtering of the said image, followed by an acceleration process that prepares the method to re-iterate if convergence has not been achieved.

For solving the equations system, we use the Least Squares QR method (LSQR) [13] since it is one of the most stable methods. The method solves the system *Af* = *g* + *w* for *w* = 0 by minimizing *min*∥*Af* − *g*∥_2_.

The non-linear filter *Soft Thresholding Filter* [14, 15] is applied after the LSQR. This filter acts as a regularization technique when we reduce the number of projections. It helps convergence, conserving not only the vertical-horizontal but also the diagonal gradient, causing the image to be sharper without losing its structure at the edges, which is of vital importance in medical CT imaging tests. Finally, the acceleration technique *Fast Iterative Shrinkage Thresholding Algorithm* (FISTA) defined in [16] is applied, which reduce the number of total iterations needed.

### Selected image filters

Considering the variety of image filtering techniques, it is necessary to limit the number of methods to test. Taking as reference the work [7], the following filtering techniques have been chosen.

#### Gaussian filter

It is the result of smoothing the image by means of a Gaussian function [17]. At the expense of reducing or eliminating noise, there is a risk of losing a large amount of detail due to the fact that the edges are not preserved. Thus obtaining a blurred and unclear image.

The Gaussian filter is applied to a 2D image as defined in (1), where *G* is the Gaussian mask with coordinates (*x*, *y*), *σ* is the parameter that defines the standard deviation. If the value of *σ* is large, the image smoothing effect will be greater. The smoothing can be done by convolutioning a window of the original image *I (x, y)* of size *w* × *h* with a Gaussian mask *G* as illustrated in the Eq (2). The filtered image is obtained by calculating the sum of products between all the pixels of the input image window and the Gaussian matrix.

$$G\left(x,y\right)=\frac{1}{2\pi \sigma^{2}}e^{-\left(x^{2}+y^{2}\right)/2\sigma^{2}}$$ (1)

$$f\left(x,y\right)=\sum_{i=0}^{w-1}\sum_{j=0}^{h-1}G\left(i,j\right)I\left(x-i,y-j\right)$$ (2)

#### Median filter

The median filter is a non-linear method [18]. It is widely used as it is very effective in eliminating noise and preserving edges. It is particularly effective in eliminating ‘salt and pepper’ noise.

The median filter works by moving through the image pixel by pixel, replacing each value with the median value of the neighboring pixels. The neighbor pattern is called “window”, which slides, pixel by pixel over the entire image.

In the limits of the image, there are no previous or subsequent values, the value of the repeated pixel itself is used in the zones that do not have values. It is also possible to fill empty spaces with zeros or ones.

#### Wiener filter

By inverse filtering, it eliminates the additive noise and inverts the blurring of the image simultaneously [18]. It considers images and noise as random variables. The objective is to find an estimate of the original image such that the mean square error between them is minimized.

The filter estimates the local mean (3) and the variance (4) around each pixel, where *η* is the local neighborhood *N* × *M* of each pixel in the image *A*. It then filters (5) pixel by pixel using these estimates, where *v*^2^ is the variance of the noise. If the noise variance is not provided, the average of all estimated local variances is used.

$$\mu =\frac{1}{NM}\sum_{n_{1},n_{2}\in \eta }a\left(n_{1},n_{2}\right)$$ (3)

$$\sigma^{2}=\frac{1}{NM}\sum_{n_{1},n_{2}\in \eta }a^{2}\left(n_{1},n_{2}\right)-\mu^{2}$$ (4)

$$b\left(n_{1},n_{2}\right)=\mu +\frac{\sigma^{2}-v^{2}}{\sigma^{2}}\left(a\left(n_{1},n_{2}\right)-\mu \right)$$ (5)

#### Bilateral filter

It is a non-linear filter and edge-preserving [19]. It replaces the value of each pixel with a weighted average of the pixels next to its location. The weighting is generally based on a normal distribution according to the values of the pixels.

The bilateral filter is defined as (6), where the normalization term (7) ensures that the filter preserves the energy of the image. *I*^filtered^ is the filtered image; *I* is the original input image to be filtered; *x* is the coordinates of the pixel to be filtered; Ω is the window centered on *x*; *f*_*r*_ is the range of the kernel to smooth the differences in intensities; *g*_*s*_ is the spatial range for smoothing coordinate differences. The last two functions can be Gaussian functions.

$$I^{\text{filtered}}\left(x\right)=\frac{1}{W_{p}}\sum_{x_{i}\in \Omega }I\left(x_{i}\right)f_{r}(∥I\left(x_{i}\right)-I\left(x\right)∥)g_{s}(∥x_{i}-x∥)$$ (6)

$$W_{p}=\sum_{x_{i}\in \Omega }f_{r}(∥I\left(x_{i}\right)-I\left(x\right)∥)g_{s}(∥x_{i}-x∥)$$ (7)

The weight *W*_*p*_ is assigned by spatial closeness and intensity difference. Consider a pixel located in (*i*, *j*) that needs to be filtered with its neighboring pixels and one of its neighboring pixels is in (*k*, *l*). The weight assigned per pixel (*k*, *l*) to eliminate the noise of the pixel (*i*, *j*) is given by (8). Here, *σ*_*d*_ and *σ*_*r*_ are smoothing parameters and *I*(*i*, *j*) and *I*(*k*, *l*) are the intensity of the pixels (i, j) and (k, l) respectively. After calculating the weights, we must normalize them with (9), where *I*_*D*_ is the intensity without noise of the pixel (*i*, *j*).

$$w\left(i,j,k,l\right)=e^{\left(-\frac{{\left(i-k\right)}^{2}+{\left(j-l\right)}^{2}}{2\sigma_{d}^{2}}-\frac{∥I\left(i,j\right)-{I\left(k,l\right)∥}^{2}}{2\sigma_{r}^{2}}\right)}$$ (8)

$$I_{D}\left(i,j\right)=\frac{\sum_{k,l}I(k,l)*w(i,j,k,l)}{\sum_{k,l}w(i,j,k,l)}$$ (9)

## Results and discussion

### Added noise

Before analyzing the behavior of the filters we must determine the value of the variance parameter that should be used to add both the Gaussian and Speckle noise. To do this, we tried adding noise with different variances from 0.0001 to 0.005. With our phantom projections for resolution 256x256 and 60 views, we determine that the best variance value for both types of noise is 0.0005. This adds enough noise to the sinogram to appreciate the results of the filtering methods but we preserve most of the relevant information. The experiments have been conducted using the Matlab R2018B functions to add noise.

### Sinogram filtering

**Fig 2:**
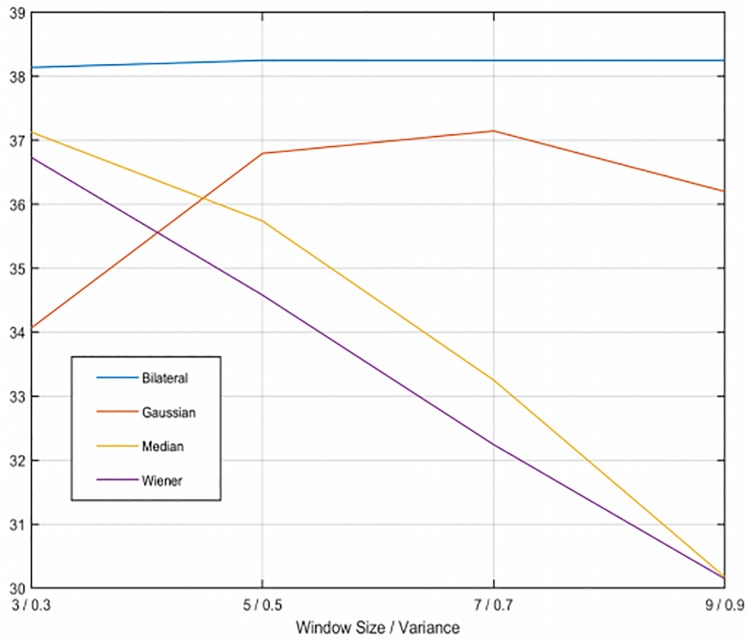
PSNR results with Gaussian noise varying the parameters. Results of filtering the sinogram with Gaussian noise using the four filters.

**Fig 3:**
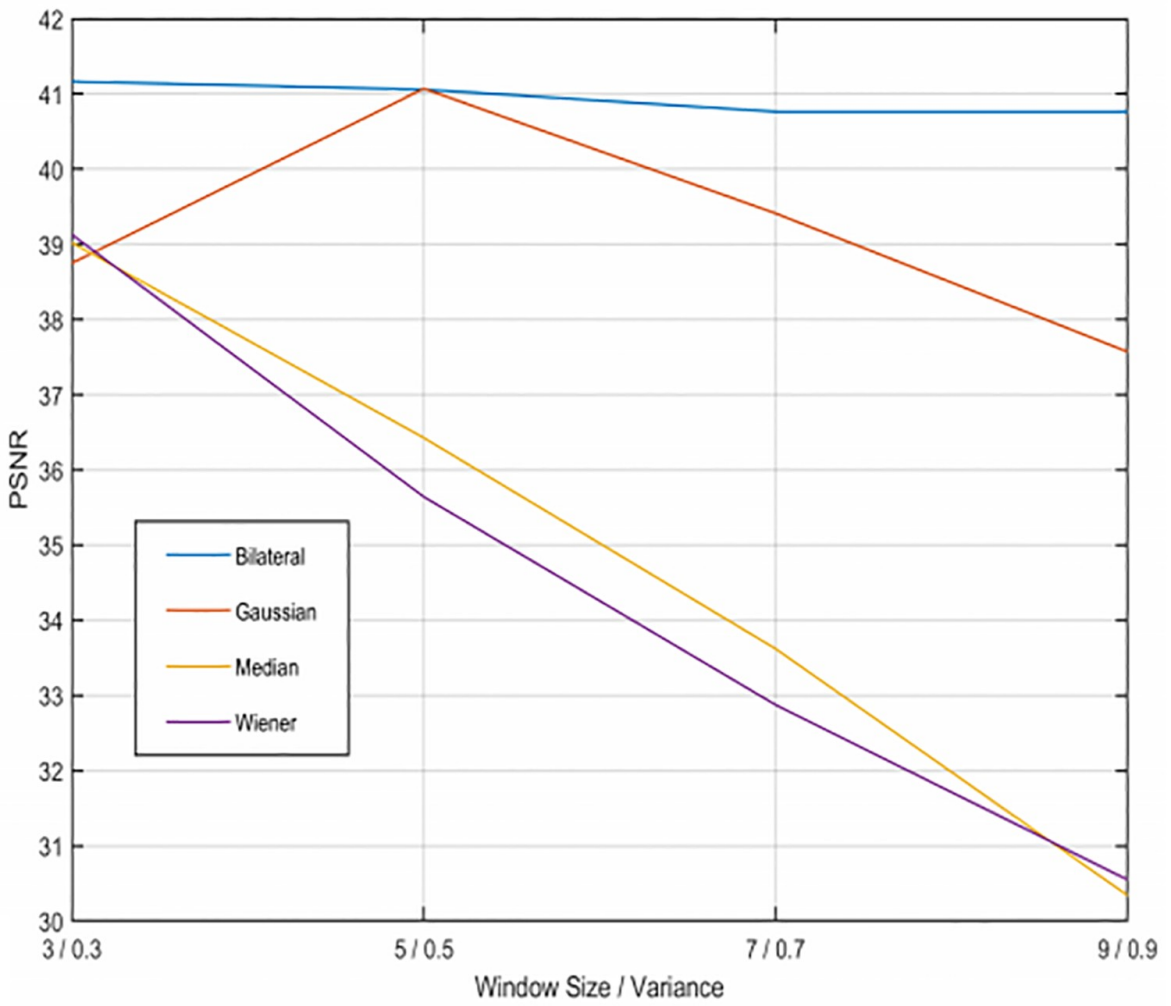
PSNR results with Speckle noise varying the parameters. Results of filtering the sinogram with Speckle noise using the four filters.

For the application of the filters, we need to consider both the variance and window size. We have applied windows from 3x3 to 9x9 pixels, and a variance value from 0.3 to 0.9. The PSNR results varying these parameters for the filters with both types of noise are presented in Figs 2 and 3. From these Figures we extract the optimum parameters for each filter.

**Fig 4:**
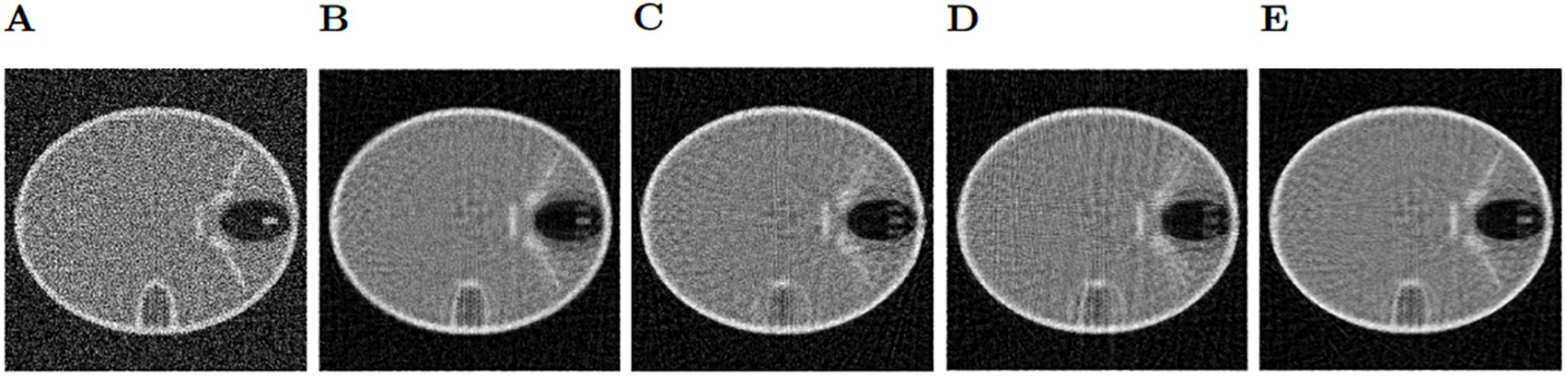
Gaussian noise reconstructions. A: Reconstructed Image with unfiltered Gaussian noise on the sinogram. B: Reconstruction using the Gaussian filter on the sinogram. C: Median filter. D: Wiener filter. E: Bilateral filter.

**Fig 5:**
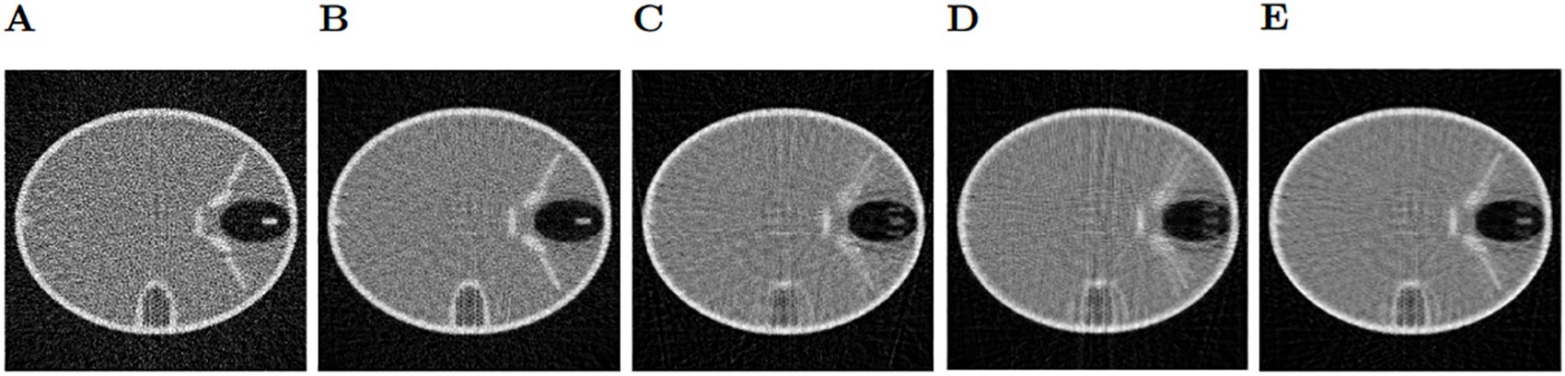
Speckle noise reconstructions. A: Reconstructed Image with unfiltered Speckle noise on the sinogram. B: Reconstruction using the Gaussian filter on the sinogram. C: Median filter. D: Wiener filter. E: Bilateral filter.

After filtering the sinogram with added Gaussian noise, we observe the bilateral filter (shown in Fig 4E) is the filtering method that achieves the best results of SSIM (Structural Similarity Index) and PSNR (Peak Signal-To-Noise Ratio) [20], in particular for 5x5 windows, although the results are very similar with every window size in this case. The second best results are obtained using the Gaussian filter (Fig 4B) with a sigma value of 0.7. Neither the median or the Wiener filter get good quality results since they alter too much the structures of the phantom. Regarding the sinograms with Speckle noise, the bilateral filter (Fig 5E) is also the best method, using a 3x3 window. Next, the Gaussian filter (Fig 5B) with a sigma value of 0.5, which gets very close to the results of the bilateral filter. As before, the Wiener and median filters don’t improve the image. It must be noted that the reconstructions shown in Figs 4 and 5 were obtained with only 200 iterations of LSQR, so the method did not reach convergence.

### Phantom filtering

**Fig 6:**
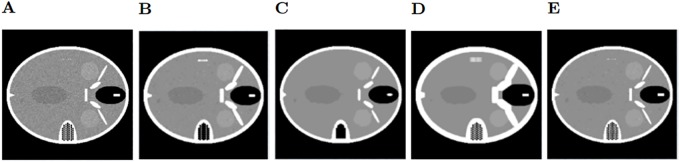
Gaussian noise filtering on the phantom image. A: Phantom with added Gaussian noise. B: Phantom filtered using Gaussian filter. C: Median filter. D: Wiener filter. E: Bilateral filter.

**Fig 7:**
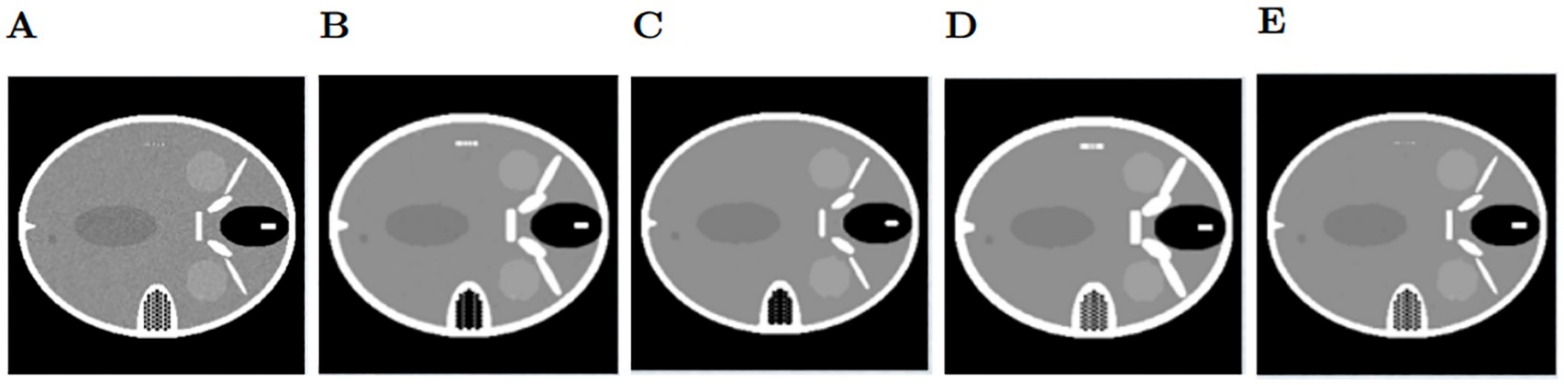
Speckle noise filtering on the phantom image. A: Phantom with added Speckle noise. B: Phantom filtered using Gaussian filter. C: Median filter. D: Wiener filter. E: Bilateral filter.

In an analogous way to the previous case, we add noise to the phantom images and then remove it using the selected filters. This can be useful when you have reconstructed images that still contain noise. The variance of the noise, as well as the window size and variance chosen for the filters are the same as before. After filtering the image with added Gaussian noise, we observe the bilateral filter (Fig 6E) is the only method that improves both the SSIM and PSNR (Tables 1 and 2), but the resulting image still has visible noise. The median filter obtains a good SSIM value since it smooths the image and the edges are better defined, but in Fig 6C we can observe that the internal structures are not well preserved, in particular the ear structure is altered. The Wiener filter (Fig 6D) alters too much the structures of the phantom and the Gaussian filter (Fig 6B) still leaves too much noise. Regarding the images with speckle noise, the bilateral filter (Fig 7E) is yet again the best method. This time, it eliminates more noise on the image, so the edges are better defined and the SSIM shows that. The other filters behave similarly to the previous case, and each of them obtain a lower PSNR than the noisy image.

**Table 1:** Phantom with Gaussian noise filtering results.

	SSIM	PSNR
Noise	0.24	50.66
Gaussian Filter	0.25	30.43
Median Filter	0.78	22.03
Wiener Filter	0.66	36.04
Bilateral Filter	0.43	57.64

**Table 2:** Phantom with Speckle noise filtering results.

	SSIM	PSNR
Noise	0.68	54.71
Gaussian Filter	0.78	23.49
Median Filter	0.93	22.01
Wiener Filter	0.82	32.61
Bilateral Filter	0.94	62.06

### Combination with iterative reconstruction

Since we have verified that the Bilateral filter is the best of the selected filters, we are going to integrate it with our iterative reconstruction technique. Thus, there are four steps in the proposed methodology to reconstruct the image from the sinograms. The first process, the LSQR technique, is used in all the combinations of the methodology and is the one that solves the equation *Af* = *g*. Then it can be combined with the regularization process, STF [14, 15], since the system matrix is rank deficient when we reduce the number of projections and it can help to avoid the streaks artifacts. Also, it can be combined with a filter that eliminates Gaussian and Speckle noise, in our case we will use the Bilateral filter. And finally, it can be combined with the FISTA [16] acceleration technique which, as we have seen in [5], considerably reduces the number of iterations. The pseudocode of this process is shown in Algorithm 1, where we can specify which of the additional steps apart from LSQR we want to perform. For LSQR, we call the function that applies *iter* iterations of the method using the matrix *A*, projections vector *g* and initial image solution *f* to obtain the new approximation of the image *f*. For this research, the Matlab *lsqr* function has been used. The Bilateral filter, STF and FISTA methods are applied to image *f*, and they overwrite it. It is worth mentioning that the computational cost of the methodology is dominated by the cost of the LSQR process.

For this experiment, we have used an abdominal CT image selected from the dataset DeepLesion [12], and projected with Joseph method for resolution 512x512 pixels. In Table 3 we show the results of the reconstruction for a different number of projections, evaluating the results using from 5 to 30 internal iterations of LSQR in steps of 5, giving a tolerance of 1e-06 (input parameters *iter* and *tolerance* in Algorithm 1). If the process does not reach convergence, the maximum number of iterations performed is 10000. For each number of projections we show the three best results, obtained when applying the STF and/or Bilateral filter and/or FISTA every 15, 10 or 5 iterations of LSQR.

**Table 3:** Reconstruction results combining the filter.

Number of Projections	LSQR iterations	STF	Bilateral filter	FISTA	SSIM	PSNR
180	15	X	X	X	0.9954	51.97
180	15	X	X		0.9849	45.94
180	15	X		X	0.9710	42.50
150	15	X	X	X	0.9935	50.17
150	15	X	X		0.9191	39.57
150	15	X			0.8854	38.69
120	10	X	X	X	0.9840	48.48
120	10	X	X		0.8950	39.01
120	10	X		X	0.8584	37.85
90	10	X	X	X	0.9762	44.53
90	10	X		X	0.7729	34.40
90	10	X	X		0.7667	34.06
60	5	X	X	X	0.9607	38.99
60	5	X		X	0.8052	35.61
60	5	X	X		0.8083	32.46
45	5	X		X	0.7355	31.74
45	5	X	X	X	0.6995	31.10
45	5	X	X		0.6378	30.01
30	5	X	X	X	0.6977	28.87
30	5	X		X	0.6363	27.68
30	5	X	X		0.5888	27.31

**Algorithm 1** Iterative reconstruction method

**Input**: A, g, iter, tolerance, maxiter, bilateral, regularization, acceleration

**Output**: f

 *Initialisation*:

1: residual = 1, *f* = 0_*N*_, totaliter = 0

2: **while** residual>tolerance **and** totaliter<maxiter **do**

3:  f = LSQR(A, g, f, iter)

4:  residual = ∥*g* − A*f∥/∥*g*∥

5:  **if** (*residual* > *tolerance*) **then**

6:   **if** (*bilateral* ⩵ *True*) **then**

7:    f = BilateralFilter(f)

8:   **end if**

9:   **if** (*regularization* ⩵ *True*) **then**

10:    f = STF(f)

11:   **end if**

12:   **if** (*acceleration* ⩵ *True*) **then**

13:    f = FISTA(f)

14:   **end if**

15:  **end if**

16:  totaliter+ = 1

17: **end while**

18: **return** *f*

Regardless of the number of projections used, the combination of the four processes is the best option, in some cases doubling the quality of the SSIM metric with respect to the second best option. For this, we conclude that the Bilateral filter is a good contribution to our method. Also, note that the more projections used, the less applications of the regularization and filtering techniques are needed for the reconstruction. This is logical since there is more information available and therefore fewer artifacts are generated. The Bilateral filter improves the quality of the image a greater amount when the number of projections is higher. However, the use of many projections is not desirable since a greater X-ray dose is induced to the patient. In some cases, when we have a high number of projections (180 and 150) the results are very similar using 10 or 15 internal iterations. Here, we opt to choose 15 since it means less applications of the additional steps and thus the reconstruction is faster.

**Fig 8:**
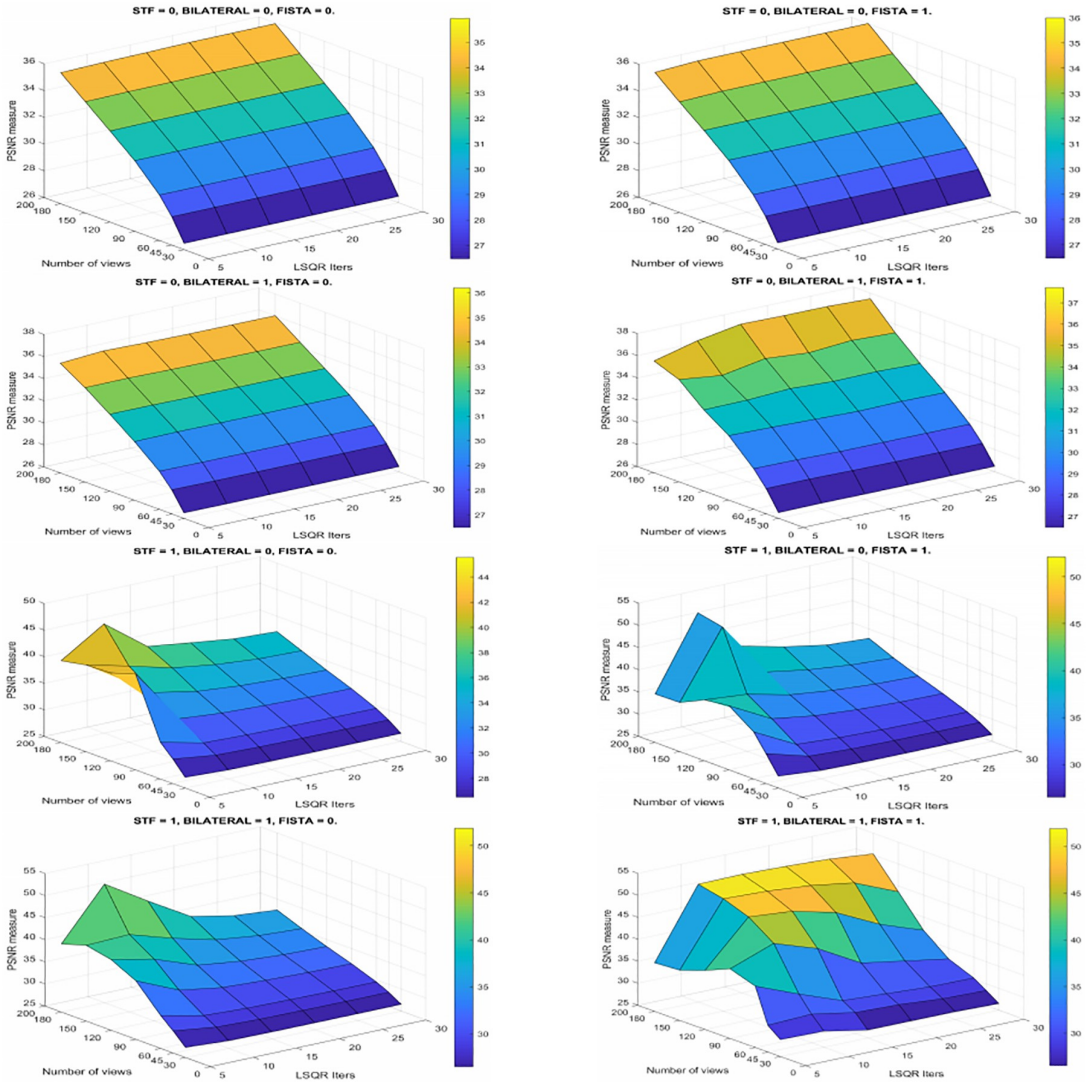
Evolution of the PSNR. Results for different number of views and internal LSQR iterations varying the combination of the additional steps.

When we perform more than 15 internal iterations, the results don’t improve in any case. We can observe this better in Fig 8 where can see the PSNR results for every number of internal iterations when we vary the number of views and combine the different STF, Bilateral and FISTA steps. Here, we observe how the PSNR increases with the number of views, but varies when we change the number of internal iterations. We also show that we obtain higher PSNR values when we use the Bilateral filter.

**Fig 9:**
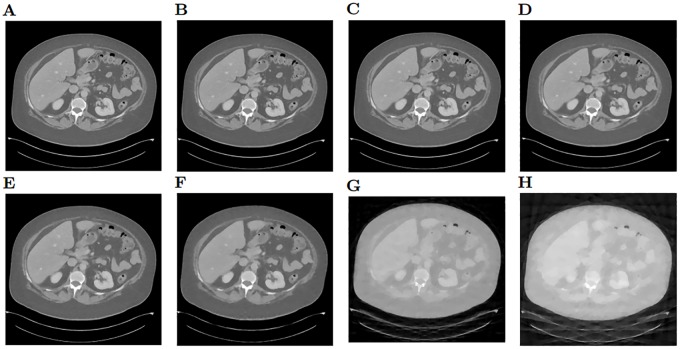
Reconstructions of an abdominal CT image. A: Reference CT image selected from dataset DeepLesion. B: Reconstruction using 180 projections. C: 150 projections. D: 120 projections. E: 90 projections. E: 60 projections. E: 45 projections. E: 30 projections.

Although the quality seems low compared with our previous studies with phantom images such as [2], since this image is much more complex, not all the reconstructions are low quality. In Fig 9 we can see the best resulting images for 180, 90, 60 and 30 projections. As we observe, the image for 30 is not good, we see streak artifacts and the internal structures are blurred and distorted. From 60 views, we start getting better images, with almost no artifacts. It is not until 90 views that we get less blurry structures and better preserved edges. From 90 to 180 projections, the images get sharper, but the internal structures we see are the same.

## Conclusion

In this work, a study of different filtering techniques on a sinogram of 60 views has been carried out. We added noise to the projections of a mathematical phantom, in order to simulate the appearance of artifacts in the acquisition of a real scanner. To evaluate the filters, it has was necessary to select a level of noise that allows us to see the effect of the filtering techniques. For this reason we chose a 0.0005 variance for both Gaussian and Speckle noise. The best reconstructions are obtained with the sinograms filtered using the bilateral method.

The filtering process has also been carried out on the reference image of the mathematical phantom with a resolution of 256x256 pixels. Again, the bilateral filter has been the one that has obtained better quality values of the filtered images with respect to the reference image for the two types of added noise. The results are significantly better for the speckle noise, improving the SSIM in a 38% and the PSNR in a 13%. As it has been possible to verify, the bilateral filter has the capacity of preserving the contours of all type of forms present in the images, at the same time as it eliminates great part of the noise.

We have also been able to test the validity of the Bilateral filter for its combination with the LSQR+STF+FISTA reconstruction method. The results for 512x512 reconstructions show that integrating the filter within the reconstruction process can improve the final quality of the images.

## Supporting information

S1 Dataset The dataset containing projection data used to reconstruct in the last section of the results in this paper is publicly available and they can be downloaded freely from the following permanent location in zenodo.org: https://doi.org/10.5281/zenodo.3603080. (DOCX)

S2 Dataset The dataset containing resulting reconstructed images in last section of the results in this paper is publicly available and they can be downloaded freely from the following permanent location in zenodo.org: https://doi.org/10.5281/zenodo.3603104. (DOCX)
