# Supplementary material for: Evaluation of image filters for their integration with LSQR computerized tomography reconstruction method
Source: PLoS One. 2020 Mar 3;15(3):e0229113. doi: 10.1371/journal.pone.0229113 (PMC7053726; doi:10.1371/journal.pone.0229113)
Supplement: S1 Dataset — (DOCX) [file pone.0229113.s001.docx]

**S1 Dataset**. <https://zenodo.org/record/3603080>
